# Image quality of late gadolinium enhancement in cardiac magnetic resonance with different doses of contrast material in patients with chronic myocardial infarction

**DOI:** 10.1186/s41747-020-00149-2

**Published:** 2020-04-03

**Authors:** Caterina Beatrice Monti, Marina Codari, Andrea Cozzi, Marco Alì, Lorenzo Saggiante, Francesco Sardanelli, Francesco Secchi

**Affiliations:** 1 Department of Biomedical Sciences for Health, Università degli Studi di Milano, Via Mangiagalli 31, 20133 Milano, Italy; 2 Department of Electronics, Information and Bioengineering, Politecnico di Milano, Via Ponzio 34/5, 20133 Milano, Italy; 3 Unit of Radiology, IRCCS Policlinico San Donato, Via Morandi 30, 20097 San Donato Milanese, Italy; 4 Unit of Diagnostic Imaging and Stereotactic Radiosurgery, C.D.I. Centro Diagnostico Italiano S.p.A., Via Saint Bon 20, 20147 Milano, Italy; 5 Postgraduate School in Radiodiagnostics, Università degli Studi di Milano, Via Festa del Perdono 7, 20122 Milano, Italy; 6 Department of Biomedical Sciences for Health, Università degli Studi di Milano, Via Morandi 30, 20097 San Donato Milanese, Italy

**Keywords:** Contrast media, Gadobutrol, Gadolinium, Magnetic resonance imaging, Myocardial infarction

## Abstract

**Background:**

Contrast-enhanced cardiac magnetic resonance (CMR) is pivotal for evaluating chronic myocardial infarction (CMI). Concerns about safety of gadolinium-based contrast agents favour dose reduction. We assessed image quality of scar tissue in CMRs performed with different doses of gadobutrol in CMI patients.

**Methods:**

Informed consent was waived for this Ethics Committee-approved single-centre retrospective study. Consecutive contrast-enhanced CMRs from CMI patients were retrospectively analysed according to the administered gadobutrol dose (group A, 0.10 mmol/kg; group B, 0.15 mmol/kg; group C, 0.20 mmol/kg). We calculated the signal-to-noise ratio for scar tissue (SNR_scar_) and contrast-to-noise ratio between scar and either remote myocardium (CNR_scar-rem_) or blood (CNR_scar-blood_).

**Results:**

Of 79 CMRs from 79 patients, 22 belonged to group A, 26 to group B, and 31 to group C. The groups were homogeneous for age, sex, left ventricular morpho-functional parameters, and percentage of scar tissue over whole myocardium (*p* ≥ 0.300). SNR_scar_ was lower in group A (46.4; 40.3–65.1) than in group B (70.1; 52.2–111.5) (*p* = 0.013) and group C (72.1; 59.4–100.0) (*p* = 0.002), CNR_scar-rem_ was lower in group A (62.9; 52.2–87.4) than in group B (96.5; 73.1–152.8) (*p* = 0.008) and in group C (103.9; 83.9–132.0) (*p* = 0.001). No other significant differences were found (*p* ≥ 0.335).

**Conclusions:**

Gadobutrol at 0.10 mmol/kg provides inferior scar image quality of CMI than 0.15 and 0.20 mmol/kg; the last two dosages seem to provide similar LGE. Thus, for CMR of CMI, 0.15 mmol/kg of gadobutrol can be suggested instead of 0.20 mmol/kg, with no hindrance to scar visualisation. Dose reduction would not impact on diagnostic utility of CMR examinations.

## Key points

- Late gadolinium enhancement is pivotal in assessing chronic myocardial infarction.
- Safety issues of gadolinium-based contrast agents advocate for dose reduction.
- Gadobutrol at 0.10 mmol/kg showed lower scar quality compared to higher doses.
- Gadobutrol at 0.15 mmol/kg provides comparable image quality to 0.20 mmol/kg.
- Gadobutrol at 0.15 mmol/kg can be suggested for assessing chronic myocardial infarction.

## Background

Coronary heart disease is one of the main causes of morbidity and mortality, especially in developed countries, where it causes around 20% of all deaths [1]. The most common presentation of coronary heart disease is myocardial infarction, which is defined as the occurrence of necrosis in the setting of myocardial ischaemia[2].

Contrast-enhanced cardiac magnetic resonance (CMR) is a multi-parametric, multi-planar imaging technique, which represents the current non-invasive standard of care for assessing cardiac volumes, function and tissue characterisation through late gadolinium enhancement (LGE) [3, 4]. The importance of LGE may be found, among other reasons, in its prognostic potential [5]. Given its capability to monitor cardiac conditions, contrast-enhanced CMR may be useful in the evaluation of patients with chronic myocardial infarction, especially when the latter is transmural and of greater clinical relevance [6, 7]. Moreover, automatic scar quantification is growing in popularity due to the increase in numbers of examinations and the development of increasingly more reliable methods [8]. Scar recognition is most often based on image characteristics of the scarred area, such as signal- (SNR) and contrast-to-noise ratio (CNR) [9].

However, especially in the latest years, concerns about the safety of gadolinium-based contrast agents (GBCA) have arisen. In addition to the well-known issue of nephrogenic systemic fibrosis [10], gadolinium deposits of yet unknown clinical relevance have been shown in the brain of patients, adults and children, who underwent repeated GBCA-enhanced magnetic resonance examinations [11, 12]. This led to a growing attention concerning the possibility to reduce GBCA doses in such examinations, provided that scar quality is not hindered.

Patients with chronic myocardial infarction hence represent a population where GBCA dose reduction would lead to a lower chance of contrast-related adverse events. At present, GBCA doses used in these patients are variable among countries and centres, usually between 0.1 (single dose) and 0.2 (double dose) mmol/kg [13]. While in some countries, such as Japan, the single dose is recommended, in most cases there are no specific indications [14]. All doses seem to provide diagnostic quality to examinations, albeit a reduction in scar visualisation corresponding to a lower contrast dosage might, for instance, hinder post-processing applications.

The purpose of our study was to analyse image quality of the scar tissue in CMR examinations performed with different GBCA doses in patients with chronic transmural myocardial infarction, to investigate the impact of gadolinium dose variation on the visibility of myocardial LGE quantified as SNR and CNR.

## Methods

### Ethical statement and study design

The local Ethics Committee approved this study (Ethics Committee of IRCCS Ospedale San Raffaele; protocol code “Cardioretro Ricerca Spontanea”; approved on September 14, 2017, and amended on July 18, 2019). This study was supported by local research funds of IRCCS Policlinico San Donato, a clinical research hospital partially funded by the Italian Ministry of Health. This research received no specific grant from funding agencies in the public, commercial, or non-profit sector. Due to the retrospective nature of this study, specific informed consent was waived.

### Study population

All patients who had undergone a contrast-enhanced CMR examination with administration of gadobutrol (Gadovist, Bayer Healthcare, Leverkusen, Germany), at our institution between March 2014 (the introduction of our newer magnetic resonance unit) and May 2018, and who were diagnosed with chronic myocardial infraction from clinical findings and CMR, were included in our study. Exclusion criteria were the presence of oedema, indicating acute phase of infarction, presence of relevant artefacts which rendered differentiation of the myocardial scar difficult, and non-transmural, thin infarcts which were either only subendocardial (≤ 50% of wall thickness) or too small (scar ≤ 10% of the myocardium), as such conditions do not allow the calculation of SNR and CNR of the scarred region [15]. Moreover, in patients with subendocardial infarction, image contrast may vary according to acquisition timing, and thus this may provide data that are not compatible with those of transmural scars [16].

Patients were then divided into three subgroups, depending on the contrast dose administered during their CMR: the first group (A) received 0.10 mmol/kg, the second (B) 0.15 mmol/kg, and the third (C) 0.20 mmol/kg. These different doses were mainly due to choices of the physicians in charge of the examination during the study period, not related to a specific patient’s condition.

### Image acquisition

All subjects were imaged using one 1.5-T whole-body magnetic resonance unit (Magnetom Aera, Siemens Healthineers, Erlangen, Germany) with 45 mT/m gradient power and an 18-channel surface phased-array coil. The examined patient was lying supine and the coil was placed over the thorax. All images were acquired with breath-holding and ECG gating.

The imaging protocol of all patients included cine and LGE sequences.

Cine images were acquired in multiple short- and long-axis planes using an ECG-triggered bright-blood steady-state free-precession pulse sequence.

LGE images were acquired after intravenous administration of 0.10, 0.15, or 0.20 mmol/kg of gadobutrol (Gadovist, Bayer Healthcare, Leverkusen, Germany) and were performed using a 2D segmented inversion-recovery fast gradient-echo sequence covering the entire left ventricle. Earlier exams utilised higher-contrast doses, which were then lowered over time. Nevertheless, the sequence for LGE imaging remained the same. The time of echo was 3.33 ms, while the time of repetition was adapted to patients’ heart rates, and inversion time was progressively modified from 260 to 330 ms, to blacken cardiac muscle; flip angle was 25°, slice thickness 8 mm, and pixel size 3.6 mm^2^. LGE images were reconstructed using magnitude reconstruction. From the R wave of the electrocardiogram, a delay period was used to ensure that image acquisition occurred in mid-diastole, when the heart is relatively motionless, therefore reducing motion artefacts. Data were acquired every other heartbeat, although in tachycardic patients data were acquired every third heartbeat, while in bradycardic patients and in patients with difficulties in breath holding acquisition was performed every heartbeat. Timing between contrast administration and acquisition of delayed enhancement scans was tailored to the contrast dose that was utilised in each case, according to literature recommendations [14].

### Image analysis

Image analysis was performed using QMass 7.6 (Medis Medical Imaging Systems, Leiden, The Netherlands). The epicardial contour of the left ventricle was manually traced for all short-axis slices at end-diastolic and end-systolic phases in cine sequences. Afterwards, a blood-thresholding technique (Mass-K mode) was applied to automatically segment myocardium and blood pool. The software then calculated end-diastolic and end-systolic volumes, both indexed and non-indexed to body surface area, myocardial mass, stroke volume, and ejection fraction.

**Fig. 1:**
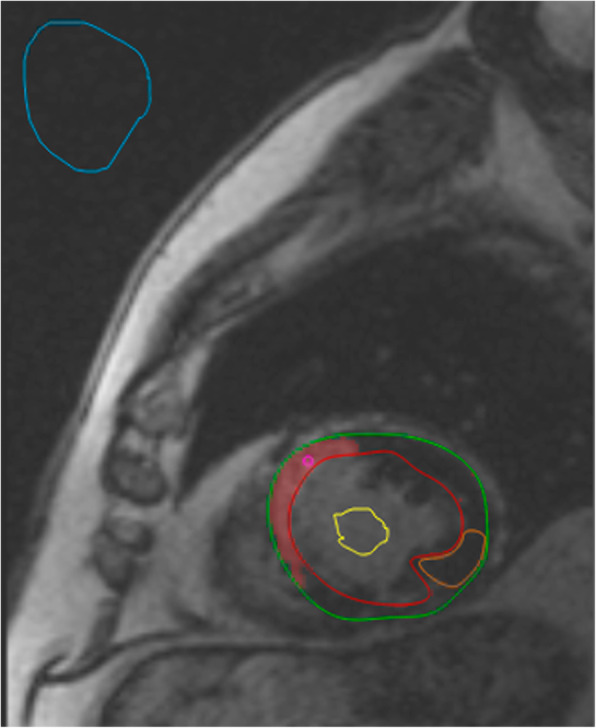
Figure showing segmentation of the scarred myocardium in a 49-year-old male patient. Scarred myocardium is shown in red and is automatically segmented at 6 standard deviations above average signal intensity. Regions of interest placement is also depicted: those in the scarred (pink) and healthy (orange) myocardium are automatically placed during scar segmentation, while the ones in the blood pool (yellow) and air (blue) are manually placed on the same image

For LGE quantification, manual segmentation of endocardium and epicardium of the left ventricle was performed in inversion recovery sequences after contrast agent injection. Then the software automatically detected the myocardial scar as being 6 standard deviations above average myocardial intensity [17]. Manual corrections were made when the software erroneously detected additional scarred areas, or when it failed to properly detect the scar. LGE was quantified as percentage over the whole myocardium. Two regions of interest were automatically placed in the scarred and healthy myocardium. An example of LGE segmentation is shown in Fig. 1.

SNR and CNR were calculated using data provided by automatic LGE quantification, namely intensities from the two ROIs automatically placed in the scarred and healthy myocardium, and two additional ROIs traced in the left ventricular blood pool and in the background air. SNR was calculated as $\mathrm{SNR}=0.655\bullet \frac{\mathrm{signal}\ \mathrm{intensity}}{{\mathrm{SD}}_{\mathrm{background}}}$ according to a study by Kaufman et al. [18], while CNR was calculated as ${\mathrm{CNR}}_{1/2}=\frac{\mid {\mathrm{signal}\ \mathrm{intensity}}_1-{\mathrm{signal}\ \mathrm{intensity}}_2\mid }{{\mathrm{SD}}_{\mathrm{background}}}$. SNR was calculated on the scar tissue (SNR_scar_), while CNR was calculated between scar tissue and remote myocardium (CNR_scar-rem_), and between scar tissue and blood (CNR_scar-blood_). Timings between contrast injection and acquisition of LGE sequences were also reported.

Subjective image quality was also analysed, using a 4-point Likert scale, defining score as follows: 0: non-diagnostic; 1: diagnostic exam, sufficient quality; 2: diagnostic exam, good quality; 3: diagnostic exam, excellent quality. The quality definition was based on the visual contrast differences between blood pool signal and LGE.

### Statistical analysis

Data were reported as median and interquartile range (IQR). Differences between groups were appraised with Kruskal-Wallis test for numerical variables, and post hoc tests when a significant difference was appraised by Kruskal-Wallis test, or Fisher *χ*^2^ tests for non-numerical variables.

Statistical analysis was performed with MATLAB R2018b (Mathworks, Natick, MA, USA), and *p* values ≤ 0.05 were considered statistically significant.

## Results

### Study population

**Fig. 2:**
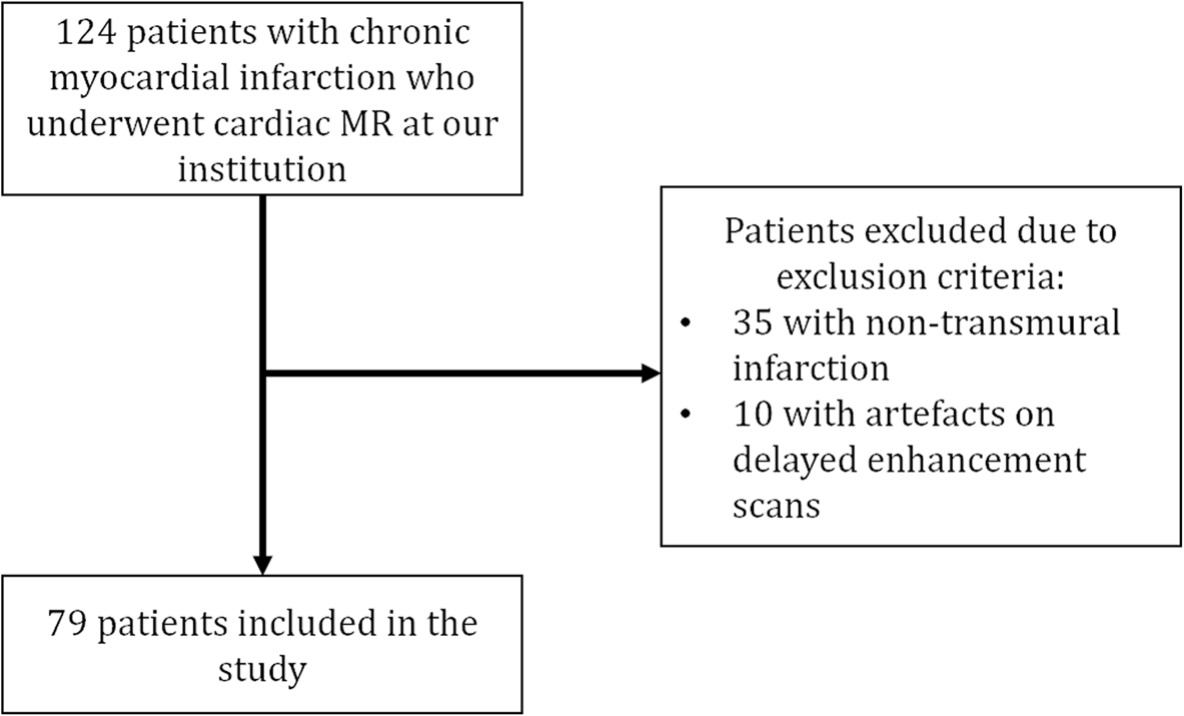
Study flowchart. Out of 124 initially retrieved patients, 35 were excluded due to their infarction not being transmural and 10 due to artefacts on late gadolinium enhancement scans regardless of the size of their infarction

Out of 124 patients who had undergone contrast-enhanced CMR at our institution, with gadobutrol as GBCA, 79 were included. The flowchart of exclusion is shown in Fig. 2. Out of the 79 included patients, 22 belonged to the group being administered 0.10 mmol/kg of gadobutrol (group A), 26 to the group being administered 0.15 mmol/kg of gadobutrol (group B) and 31 to the last group, which was administered 0.20 mmol/kg of gadobutrol (group C). There were no significant differences in either age or sex among the three groups (*p* ≥ 0.300). Group demographics are summarised in Table 1.

**Table 1:** Demographics, left ventricular function and volume, and scar data from the three study subgroups

	Group A	Group B	Group C	*p*
Number	22	26	31	−
Age (years)	68 (58–71)	62 (51–72)	60 (51–68)	0.300
Males (%)	95	92	90	0.811
LV EDVi (ml/m^2^)	94 (75–118)	93 (73–107)	100 (80–126)	0.319
LV ESVi (ml/m^2^)	58 (31–77)	56 (46–74)	63 (46–87)	0.472
LV SV (ml)	66 (46–78)	69 (60–86)	70 (62–83)	0.410
LV EF (%)	38 (28–46)	38 (31–47)	37 (30–45)	0.800
LV Mi (g/m^2^)	89 (81–116)	91 (77–114)	92 (77–102)	0.961
LGE (%)	32.5 (21.7–38.1)	30.9 (23.0–42.4)	31.1 (25.5–44.0)	0.594

*EDVi* End-diastolic volume indexed to body surface area, *EF* Ejection fraction, *ESVi* End-systolic volume indexed to body surface area, *LGE* Percentage of scar represented as late gadolinium enhancement over the myocardial mass, *Mi* Myocardial mass index, *SV* Stroke volume. Kruskal-Wallis test was used

The median acquisition time of LGE sequences was 9 min (IQR 8–13 min) for group A, 14 min (IQR 9–17 min) for group B and 17 min (IQR 14–20 min) for group C. Acquisition time showed a significant difference (*p* < 0.001) among groups; in particular, it did not differ between group A and group B (*p* = 0.105), but was shorter in group B than group C (*p* < 0.018), and shorter in group A than in group C (*p* < 0.001).

### Cardiac morphology and function

Left ventricular volumetric and functional data are reported in Table 1, along with myocardial scar burden quantified as percentage of scar tissue volume over the whole left ventricular volume. There were no significant differences in volumetric, functional or scar data.

### Image quality

**Fig. 3:**
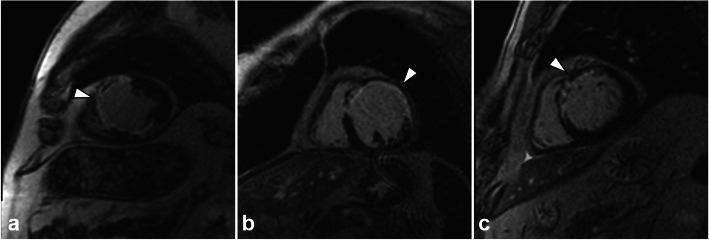
Inversion recovery sequences for late gadolinium enhancement performed using 0.10 (**a**), 0.15 (**b**), or 0.20 (**c**) mmol/kg of gadobutrol, in male patients of 76, 54, and 49 years of age, respectively, matched for percentage infarct size

Images of LGE in patients belonging to the three different groups are shown in Fig. 3.

SNR_scar_ was 46.4 (IQR 40.3–65.1) in group A, 70.1 (IQR 52.2–111.5) in group B, and 72.1 (IQR 59.4–100.0) in group C. There was a significant difference in SNR_scar_ among groups (*p* = 0.002), in particular SNR_scar_ in group A was lower than both that of group B (*p* = 0.013) and group C (*p* = 0.002), while there was no significant difference in SNR_scar_ between group B and group C (*p* = 0.884).

CNR_scar-rem_ was 62.9 (IQR 52.2–87.4) in group A, 96.5 (IQR 73.1–152.8) in group B, and 103.9 (IQR 83.9–132.0) in group C. There was a significant difference in CNR_scar-rem_ among groups (*p* < 0.001), in particular CNR_scar-rem_ in group A was significantly lower than both that of group B (*p* = 0.008) and group C (*p* = 0.001), while there was no significant difference in CNR_scar-rem_ between group B and group C (*p* = 0.871).

CNR_scar-blood_ was 25.5 (IQR 14.4–35.0) in group A, 32.7 (IQR 17.9–60.8) in group B, and 29.6 (IQR 18.2–53.5) in group C. There were no significant differences in CNR_scar-blood_ among groups (*p* = 0.335).

**Fig. 4:**
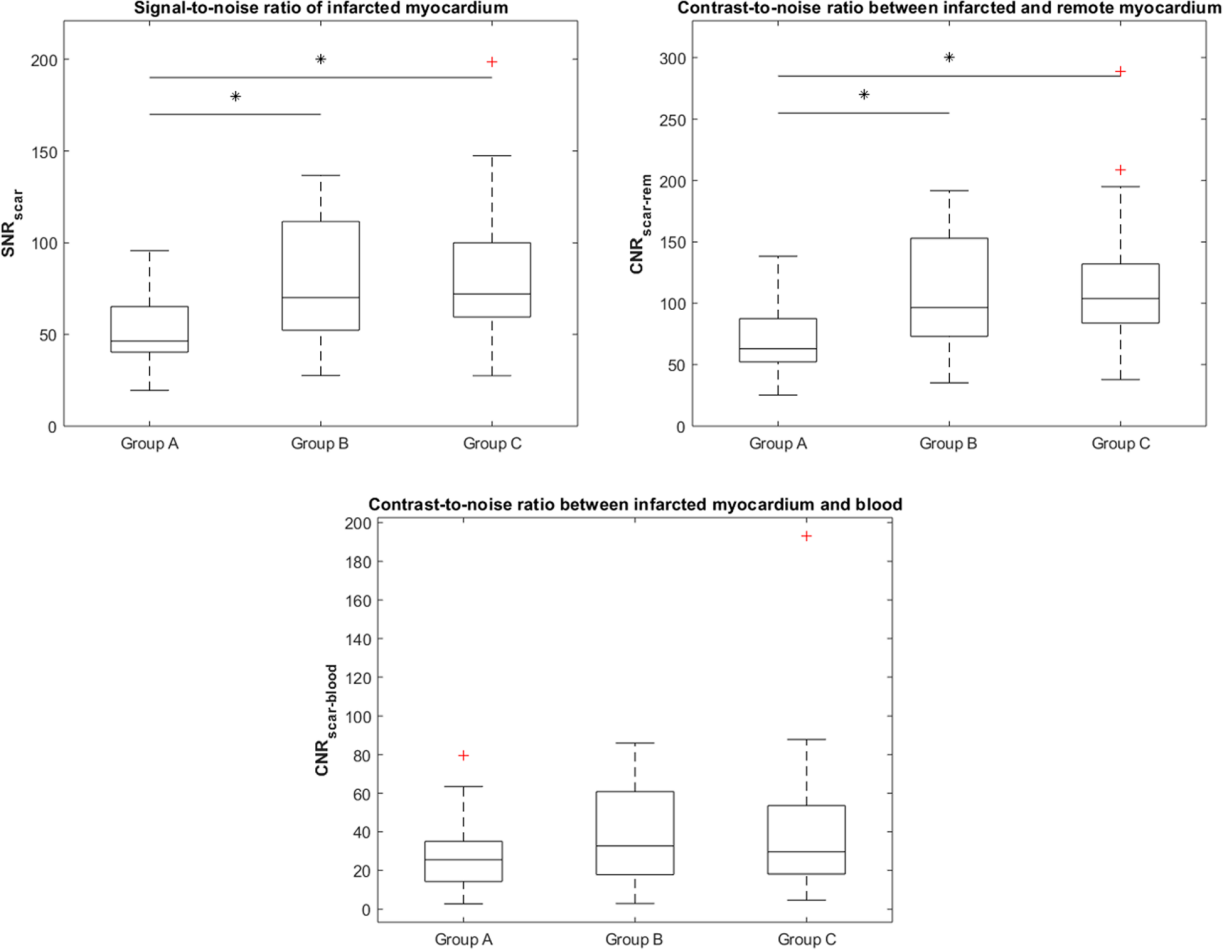
Box plots of signal-to-noise ratio of the scarred myocardium (SNR_scar_), contrast-to-noise ratio between infarcted and remote myocardium (CNR_scar-rem_), and contrast-to-noise ratio between infarcted myocardium and blood (CNR_scar-blood_) in the three groups being administered 0.10 (group A), 0.15 (group B), and 0.20 (group C) mmol/kg of gadobutrol. Significant differences between groups are indicated with an asterisk (*), and red crosses (+) indicate outliers. In particular, SNR_scar_ was lower in group A (46.4 IQR 40.3–65.1) than in both group B (70.1 IQR 52.2–111.5, *p* = 0.013) and group C (72.1 IQR 59.4–100.0, *p* = 0.002), and CNR_scar-rem_ was lower in group A (62.9 IQR 52.2–87.4) than in both group B (96.5 IQR 73.1–152.8, *p* = 0.008) and group C (103.9 IQR 83.9–132.0, *p* = 0.001). There were no other significant differences in SNR_scar_, CNR_scar-rem_, or CNR_scar-blood_ (*p* ≥ 0.335)

Box plots of SNR_scar_, CNR_scar-rem_, and CNR_scar-blood_ across the three groups are depicted in Fig. 4, and data are reported in Table 2.

**Table 2:** Image quality and differences among the three groups according to the dose of gadobutrol used for late gadolinium enhancement

	Group A	Group B	Group C	*p* value (global)	*p* value (A *versus* B)	*p* value (B *versus* C)	*p* value (A *versus* C)
SNR_inf_	46.4 (40.3–65.1)	70.1 (52.2–111.5)	72.1 (59.4–100.0)	0.002*	0.013*	0.884	0.002*
CNR_scar-rem_	62.9 (52.2–87.4)	96.5 (73.1–152.8)	103.9 (83.9–132.0)	< 0.001*	0.008*	0.871	0.001*
CNR_scar-blood_	25.5 (14.4–35.0)	32.7 (17.9–60.8)	29.6 (18.2–53.5)	0.335	−	−	−

Group A received 0.10 mmol/kg, group B 0.15 mmol/kg, and group C 0.20 mmol/kg of gadobutrol. *CNR*_*scar-blood*_ Contrast-to-noise ratio between myocardial scar and blood, *CNR*_*scar-rem*_ Contrast-to-noise ratio between scarred and remote healthy myocardium, *SNR*_*inf*_ Signal-to-noise ratio of the myocardial scar. Kruskal-Wallis and Fisher *χ*^2^ tests were used

*Indicates statistical significance

Concerning subjective image quality, no exams were non-diagnostic (Likert score 0), 7 exams displayed sufficient quality (Likert score 1), 24 exams good quality (Likert score 2), and 48 exams excellent quality (Likert score 3). In group A, 4 exams displayed sufficient quality, 7 good quality, and 11 excellent quality. In group B, 3 exams displayed sufficient quality, 7 good quality, and 16 excellent quality. In group C, 10 exams displayed good quality and 21 excellent quality. There were no significant differences in subjective image quality among groups (*p* = 0.250)

## Discussion

The issue of GBCA dose reduction has become crucial in the last few years [19]. Among patients who undergo contrast-enhanced CMR, one of the main groups is represented by patients with chronic myocardial infarction, especially when the infarct is transmural and of greater clinical relevance [20]. In this study, we wished to ascertain whether lower GBCA doses resulted in lower scar image quality, or if there was room for dose reduction while preserving scar visibility. Even lower GBCA doses guarantee diagnostic quality; however, especially given the rise of automatic post-processing methods, it may be important to preserve the highest possible scar discernment to ensure images can be utilised for such purposes. In fact, the quantification of LGE using standard deviations may be influenced by SNR and CNR, as lower SNR and CNR may signify that background noise has a higher impact on intrinsic signal intensity variations, and this may lead to less accurate scar detection, for instance using standard deviation-related systems.

Acquisition time was optimal in all groups, never exceeding 30 min as recommended by the literature [21]. Moreover, the differences in acquisition timings reflect the recommendations to obtain adequate image contrast according to the dose of contrast agent used [14]. Among our study groups, there were no significant differences in demographics or volumetric or functional left ventricle data and scar percentage over the whole myocardium. This would imply that none of these variables should have influenced the results of our research.

Concerning scar visibility, a lower SNR_scar_ (see Table 2) in group A than in both group B and group C could be due to the fact that a 0.10 mmol/kg GBCA dose was not sufficient to enhance the scarred myocardium in the same way as the two other doses, even though timing was appropriate for LGE (median 9 min, IQR 8–13 min) [22]. This hypothesis is also supported by a lower CNR_scar-rem_ (see Table 2) in group A than in both group B and group C. CNR_scar-blood_ showed no differences (see Table 2) between group A and group B, in accordance with our hypothesis, since both the scarred myocardium and blood are enhanced by the same contrast dose and are still enhanced at the time of LGE acquisition. SNR_scar_ was not significantly different between group B and group C, neither did CNR_scar-rem_ and CNR_scar-blood_, suggesting that image quality between the two doses of 0.15 mmol/kg and 0.20 mmol/kg of gadobutrol is comparable.

Our results concerning SNR and CNR were not always similar to those obtained by other authors using the same doses of gadobutrol. At 0.10 mmol/kg, our SNR_blood_ was lower than that obtained for by De Cobelli et al. [23] using gadobutrol 0.10 mmol/kg on a group of patients with mixed pathologies exhibiting LGE. Our CNR_scar-rem_ was on average slightly lower than theirs but overlapping to a certain degree due to the wide range of distributions; conversely, our CNR_scar-blood_ was higher. Their method of calculating SNR was equal to ours except for the lack of the 0.655 adjusting factor which would indeed lower our SNR compared to theirs. Their method of calculating CNR was equal to ours. Concerning 0.15 mmol/kg, both CNR_scar-rem_ and CNR_scar-blood_ were higher than those obtained by Durmus et al. [24] utilising gadobutrol at 0.15 mmol/kg with a 15-min delay to LGE scan. Durmus et al. used the same method for calculating CNR as our study. However, we should consider that our study only included transmural infarctions, while these authors did not exclude patients by scar size. Concerning the comparison of objective image quality parameters, while studies have assessed the differences between different contrast agents at different doses [25, 26], to our knowledge none have yet compared different gadobutrol doses.

This study has some limitations, the first being its retrospective design. Results refer to the specific sequence for LGE used at our centre, and to gadobutrol. However, fast inversion-recovery gradient-echo sequences are widely used in clinical practice, and our timings for LGE are aligned to recommendations [21]. On the other hand, gadobutrol is commonly used in CMR [13], it has a double concentration (1.0 M) in comparison with all other vascular/interstitial GBCAs and exhibits an r1-relaxivity relatively higher. However, the double molarity should not impact on LGE findings (obtained after about 10 min after injection), especially concerning SNR and CNR_scar-rem_, as observed by Wildgruber et al. [26], while the clearance of each single GBCA might impact on CNR_scar-blood_. Conversely, since the relatively higher relaxivity of gadobutrol may have positively impacted objective image quality of LGE imaging, as also reported by Schlosser et al. [27], the results obtained for gadobutrol may not be generalizable to GBCAs with a lower relaxivity. Another potential limitation could be posed by the variability of the placement of the regions of interest in the different areas. Nevertheless, the two regions of interest in scarred and healthy myocardium, which were the ones that could carry more issues, were automatically placed by the scar quantification software, and the ones in the air and the blood pool, which were hand-drawn, brought less difficulties. One further limitation, related to the retrospective nature of the study, is represented by the method used for SNR and CNR calculation. In fact, with the only availability of LGE sequences for the assessment of such parameters, the lone viable method for SNR and CNR calculation depended on the use of ROIs placed on the desired structures and background. However, this method has shown to provide the highest variability on SNR in a study by Dietrich et al. [28]. An ideal method for SNR and CNR calculation would perhaps be the one presented by Holtackers et al. [29], who utilised subsequent acquisitions of the same sequence using different inversion times. Nevertheless, we utilised the same sequence for all patients, thus variations in SNR and CNR should be of a systematic nature, thus preserving statistical significance of the observed differences.

In conclusion, results from our study suggest that, while 0.10 mmol/kg of gadobutrol provides inferior scar image quality of CMI than 0.15 and 0.20 mmol/kg, the last two dosages seem to provide similar LGE. In view of a global trend of standardisation and reduction of GBCA doses, 0.15 mmol/kg of gadobutrol could be suggested instead of 0.20 mmol/kg, with no hindrance to image quality. Further studies should be conducted to evaluate whether lower GBCA dosages provide a high enough scar quality for clinical evaluations. This would pave the way for further GBCA dose reduction which may impact on image quality, but not on diagnostic utility of CMR examinations.

## Data Availability

All data obtained or analysed during this study are included in this published article.

## Abbreviations

CMR: Cardiac magnetic resonance
CNR: Contrast-to-noise ratio
CNR_scar-blood_: Contrast-to-noise ratio between scar tissue and blood
CNR_scar-rem_: Contrast-to-noise ratio between scar tissue and remote myocardium
GBCA: Gadolinium-based contrast agents
IQR: Interquartile range
LGE: Late gadolinium enhancement
SNR: Signal-to-noise ratio
SNR_scar_: Signal-to-noise ratio of scar tissue
